# Composites of a Polypropylene Random Copolymer and Date Stone Flour: Crystalline Details and Mechanical Response

**DOI:** 10.3390/polym13172957

**Published:** 2021-08-31

**Authors:** Amina Benarab, Enrique Blázquez-Blázquez, Rachida Krache, Rosario Benavente, María L. Cerrada, Ernesto Pérez

**Affiliations:** 1 LMPM, Département de Génie des Procédés, Faculté de Technologie, Université Ferhat Abbes, Sétif 19000, Algeria; lamiamina19@gmail.com (A.B.); rachida2000fr@yahoo.fr (R.K.); 2 Instituto de Ciencia y Tecnología de Polímeros (ICTP-CSIC), Juan de la Cierva 3, 28006 Madrid, Spain; enrique.blazquez@ictp.csic.es (E.B.-B.); rbenavente@ictp.csic.es (R.B.); mlcerrada@ictp.csic.es (M.L.C.)

**Keywords:** polypropylene random copolymer, date stone flour, sorbitol derivative, X-ray diffraction, differential scanning calorimetry, microhardness

## Abstract

Several composites were prepared based on a polypropylene random copolymer (PPR) and different amounts of date stone flour (DSF). This cellulosic fiber was silanized beforehand in order to reduce its hydrophilicity and improve the interfacial adhesion with the polymer. Other composites were also obtained, including a sorbitol derivative as an effective nucleant. Films made from these composites were prepared using two different thermal treatments, involving slow crystallization and rapid cooling from the melt. Scanning electron microscopy was used to evaluate the morphological features and the DSF particle dispersion within the PPR matrix. X-ray diffraction experiments and differential scanning calorimetry tests were employed to assess the crystalline characteristics and for the phase transitions, paying especial attention to the effects of the DSF and nucleating agent on PPR crystallization. An important nucleation ability was found for DSF, and evidently for the sorbitol derivative. The peak crystallization temperature upon cooling was considerably increased by the incorporation of either the nucleant or DSF. Additionally, a much higher proportion of orthorhombic crystals developed in relation to the monoclinic ones. Moreover, the mechanical responses were estimated from the microhardness experiments and significant improvements were found with increasing DSF contents. All of these findings indicate that the use of silanized DSF is a fairly good approach for the preparation of polymeric eco-composites, taking advantage of the widespread availability of this lignocellulosic material, which is otherwise wasted.

## 1. Introduction

In recent years, there have been growing concerns regarding sustainability and preservation of the environment. These issues, together with the reduction of petroleum sources and the new and more stringent conservational regulations, have increased the need for new environmentally friendly materials that will contribute to achieving a circular economy.

One of the possibilities in this direction is the use of so called eco-composites or green composites containing natural fibers [1,2,3,4,5,6,7,8]. These fibers are naturally abundant, easily accessible, and there are many types, meaning they are valuable alternative resources, with the additional advantage that they require little energy for their eventual processing [8]. They represent, therefore, valuable alternatives to the use of more traditional fillers, such as calcium carbonate, silica, glass fiber, and carbon nanotubes [1,9,10,11,12,13], which involve additional difficulties in recycling.

Those eco-composites, however, have certain drawbacks, mainly related to the great variations in their properties (depending on the kind of fiber, environmental conditions, and processing procedures [8,9]), their poorer processability, and because they may also require particular compatibilization methods, depending on the polymeric matrix employed [1,9]. Using specific compatibilizers or suitable modification of the filler by appropriate chemical treatments will overcome some of these drawbacks. These chemical treatments aim to reduce the hydrophilicity of the cellulosic fibers and to improve the interfacial adhesion with the polymeric component.

One of the fillers of plant origin is date stone flour (DSF), a lignocellulosic material resultant from date fruit, which is readily obtainable in North African regions. Only a few studies have reported on polymer composites containing DSF [9,14,15,16,17], which discussed the compatibilization problems mentioned above and employed a compatibilizer.

Regarding the polymer matrix, polypropylene, PP, is an extensively used polyolefin material, due to its good price–performance balance. This feature makes it an attractive offering, with uses in a number of sectors, as well as mass consumption and engineering applications [18]. The considerable development of PP has mainly been due to the different structures and morphologies that it can generate by altering synthesis and processing conditions via the presence of foreign surfaces in the PP matrix [19,20].

Increasing interest has been devoted in recent years to composites of PP and natural fibers, addressing various aspects of fiber reinforcement, the different fiber properties, the incorporation and treatment of the fillers, and nucleation details, among others [21,22,23,24,25,26,27].

Among PP polymers, there is a special classed named polypropylene random copolymer (PPR), which according to standard ASTM F2389, is based on propylene and at least one comonomer, with propylene being above 50% of the composition. The most usual comonomers are ethylene or 1-butene, and their presence involves certain decreases of the glass transition and melting temperatures and of the crystallinity and rigidity in relation to the properties exhibited by PP homopolymers [28,29,30,31].

The aims of this study are the preparation, characterization, and preliminary evaluation of the properties of various composites based on a PPR as the polymer matrix and containing different amounts of DSF as the plant fiber filler. Moreover, the effects of the addition of a sorbitol derivative, as a nucleant and clarifying agent, are also investigated. The characterization process includes scanning electron microscopy (SEM) to evaluate the filler particle dispersion in the PPR matrix, as well as X-ray diffraction experiments and differential scanning calorimetry (DSC) tests to assess morphological and crystalline features and phase transitions, paying especial attention to the effects of the DSF and nucleant on the crystallization capability. In addition, microhardness experiments are carried out to preliminarily estimate their mechanical responses.

## 2. Materials and Methods

### 2.1. Materials and Chemicals

The polymer used in this investigation was a polypropylene–ethylene random copolymer (PPR), synthesized by a Ziegler-Natta catalyst (from Repsol, Madrid, Spain) and with an ethylene content of 3.8 wt.%. The molar masses, measured by gel permeation chromatography, were M_w_ = 415 Kg/mol and M_n_ = 97 Kg/mol, with a polydispersity ratio of M_w_/M_n_ = 4.3.

The sorbitol derivative incorporated into PPR was Millad 3988 (1,3:2,4bis (3,4dimethylbenzylidene) sorbitol), which was used as a nucleant and clarifying agent, supplied by Milliken Chemical.

The cellulosic fiber used was date stone, obtained from Deglat-Ennour (Wargla, Algeria), the chemical composition of which was determined according to the Technical Association of the Pulp and Paper Industry (TAPPI) test [32,33]. The obtained results are presented in Table 1.

Vinyltrimethoxysilane was selected for the modification of the DSF particles. It was purchased from Aldrich and used as received.

### 2.2. Chemical Modification of Date Stone

To enhance the interfacial adhesion with the polymer, the silanization method was selected from the different chemical treatments commonly used to reduce the hydrophilicity of cellulosic fibers; thus, the chemical modification of date stone, leading to the date stone flour (DSF), was performed using silane [34]. For this, 100 g of the date stone flour was incorporated into a methanol/water (90:10) (*w*/*w*) mixture by application of continuous stirring using a magnetic bar for 12 h and at room temperature (25 °C). Afterwards, the resultant mass was filtered and dried at 80 °C. A dry pristine flour was then obtained. On the other hand, 5% vinyltrimethoxysilane was dissolved in a similar methanol/water mixture (90:10) (*w*/*w*) as before, while the pH was adjusted to 4 by adding acetic acid by applying continuous stirring for 10 min and at a temperature of 25 °C. The previously dried flour was immersed in the prepared solution and stirring was applied for 12 h at 25 °C. Next, it was filtered and dried at 80 °C for 12 h and a dry silanized date stone flour was obtained. In the following, and for simplicity, DSF will be used to refer to this modified date stone flour.

### 2.3. Preparation of Composites and Films

The composites of PPR and DSF, with and without nucleating agent (Millad 3988), and with the presence of 0.1 wt.% of antioxidant additives (Irganox 1010 and Irgafos 168) to prevent thermo-oxidation of PPR, were prepared using a Brabender, with a screw speed of 40 rpm, mixing time of 12 min, at a temperature of 180 °C.

The formulations for different composites and the sample code nomenclature are shown in Table 2.

The films used for subsequent characterization were obtained from the different composites by compression molding in a Collin press between hot plates at 180 °C and with a pressure of 25 MPa for 3 min. Then, two different thermal treatments were applied. The first was a slow cooling from the melt to room temperature at the inherent cooling rate of the press after the power was switched off (cooling rate around 1 °C/min), while maintaining the pressure constant at 25 MPa. This thermal treatment was called S. The second procedure involved a relatively rapid cooling (around 100 °C/min) from the melt to room temperature by refrigerating the press plates with cooling water. This second film treatment was named Q. The film thickness was around 250 µm.

### 2.4. Scanning Electron Microscopy

The morphology of the samples was analyzed at room temperature in an PHILIPS XL30 ESEM environmental scanning electron microscope (Leuven, Belgium) operating at 25 kV, using a secondary electron (SE) detector. The samples were cryofractured using liquid nitrogen before analysis.

### 2.5. X-ray Diffraction Experiments

Conventional wide-angle X-ray diffraction patterns were recorded in reflection mode using a Bruker D8 Advance diffractometer provided with a PSD Vantec detector (from Bruker, Madison, Wisconsin). Cu Kα radiation (λ = 0.15418 nm) was used, operating at 40 kV and 40 mA. The parallel beam optics were adjusted using a parabolic Göbel mirror with a horizontal grazing incidence Soller slit of 0.12° and an LiF monochromator. The equipment was calibrated with different standards, namely Al_2_O_3_ (Corundum) and Cr_2_O_3_.

The X-ray crystallinity was determined by subtracting the corresponding amorphous component taken from the profile of a totally amorphous elastomeric PP sample [35,36].

### 2.6. Differential Scanning Calorimetry

The phase transitions were studied by DSC in a TA Instruments Q100 calorimeter connected to a cooling system and under nitrogen purge. Different cooling rates from the melt were tested, ranging from 40 to 1 °C/min. The application of higher cooling rates was not possible since the calorimeter loses temperature control before the crystallization exotherm occurs. The subsequent melting curves were registered in the temperature range of −20 to 180 °C at a scanning rate of 20 °C/min. The sample weights were about 5 mg. The values of T_c_ and T_m_ were obtained from the peak maxima of the exothermic and endothermic events, respectively.

For the determination of the DSC crystallinity, a value of 160 J/g [37,38] was used as the enthalpy of fusion of a perfectly crystalline material.

### 2.7. Microhardness

The microhardness (MH) measurements were performed with a Vickers indentor [39]. The MH values were estimated from the following expression [40]:

MH = 2 sin 68° (P/d^2^) (1)

where P is the contact load (in N) and d is the length of the diagonal of the indentation surface (in mm). Diagonals were measured in the reflected light mode within 30 s of load removal using a digital eyepiece equipped with a Leitz computer–counter–printer (RZA-DO). All of the measurements were carried out with a load of 0.981 N and a contact time of 25 s at room temperature. Five measurements were performed in different parts of the film for each specimen. The mean values of MH and their corresponding standard deviation were then estimated.

## 3. Results

### 3.1. Scanning Electron Microscopy

**Figure 1:**
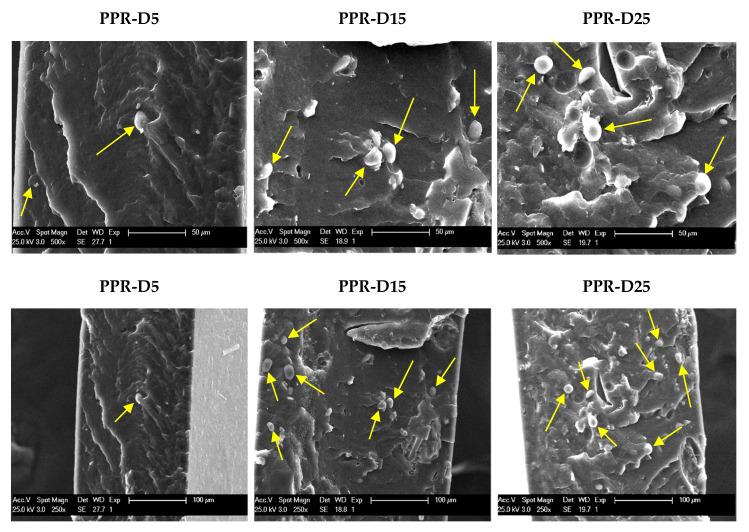
SEM images at two magnifications for the indicated samples. Some of the DSF particles are marked by yellow arrows.

Figure 1 shows the SEM images obtained for the three PPR composites at two different magnifications. The images taken from the fracture surface reveal proper dispersion of the particles even with high loads of DSF. The spherical shape of the particles can also be deduced, with an average size of around 10 microns. Several voids can be seen after sample fracture, which could indicate inefficient interactions between the modified microparticles and the polymeric matrix. The results showing the great nucleating effect of DSF (see below) are, however, indicative of either good dispersion or suitable interactions.

### 3.2. X-ray Diffraction

**Figure 2:**
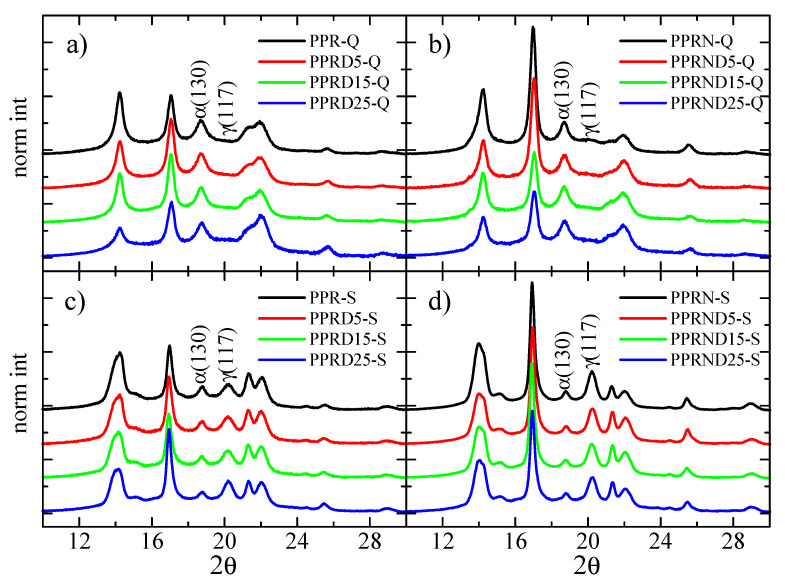
X-ray diffractograms for the different samples: (**a**) PPR-Q samples; (**b**) PPRN-Q samples; (**c**) PPR-S samples; (**d**) PPRN-S samples. The curves are displaced vertically for clarity.

The X-ray diffractograms for the different samples under the two thermal treatments are shown in Figure 2, indicating the presence of more than one crystalline form. The interesting polymorphism exhibited by isotactic PP is well documented, which depends mainly on the polymerization method, molecular features, thermal history, and on the use of different nucleants. Three main polymorphs, all showing the 3_1_ helix conformation, are reported in [41,42,43,44,45,46], named as α, β, and γ modifications. Moreover, a mesomorphic phase can be obtained under fast quenching conditions [20,41,42,43,44,47].

Additionally, a new trigonal lattice was recently reported for isotactic propylene copolymers with high comonomer contents of 1-hexene or 1-pentene, and also in terpolymers with both 1-pentene and 1-hexene or with 1-pentene and 1-heptene as the comonomers [48,49]; all these copolymers and terpolymers are prepared with metallocene catalysts.

Among those polymorphs, the α-form, with a monoclinic unit cell, is the thermodynamically most stable crystal modification of isotactic PP. On the other hand, the γ-form, with an orthorhombic structure, is favored by low molecular weights and in copolymers with α-olefins, as well as for samples polymerized by metallocenic catalysts.

Figure 2a shows the diffractograms corresponding to the rapidly cooled specimens of virgin PPR and to the three composites with DSF. The neat PPR-Q sample exhibits the diffractions typical of the α-form, with the most prominent ones appearing at values of 2θ = 14.1°, 16.9°, 18.7°, 21.1°, 21.8°, and 25.6°, corresponding to crystalline planes of (110), (040), (130), (111), (131)/(041), and (060), respectively. In principle, only slight changes occur in the diffractograms due to the addition of DSF.

The situation is different for the slowly cooled samples, as can be observed in Figure 2c; diffractions characteristic of the γ polymorph can be clearly observed, together with those of the α modification. Most of diffractions for the γ crystals almost overlap completely in the 2θ position with those from the α crystallites, with the clear exception of diffraction γ (117), which appears to be well isolated at 2θ = 20.2°.

In fact, the relative percentage of the γ polymorph can be determined [43] by using the following equation:

f_γ_ = 100 ^.^ I_γ(117)_/(I_γ(117)_ + I_α(130)_) (2)

where I_γ(117)_ and I_α(130)_ are the intensities of the diffractions γ (117) and α (130) appearing at 2θ angles of 20.2° and 18.7°, respectively. The results for the γ fraction are discussed below.

Regarding the samples nucleated with Millad, the corresponding diffractograms for the two thermal treatments, Q and S, are presented in Figure 2b,d, respectively. No important differences were observed for the Q samples in relation to the profiles for the non-nucleated samples. Diffractograms of the slowly cooled S samples, however, indicated that the γ content had increased in these PPRN specimens.

**Figure 3:**
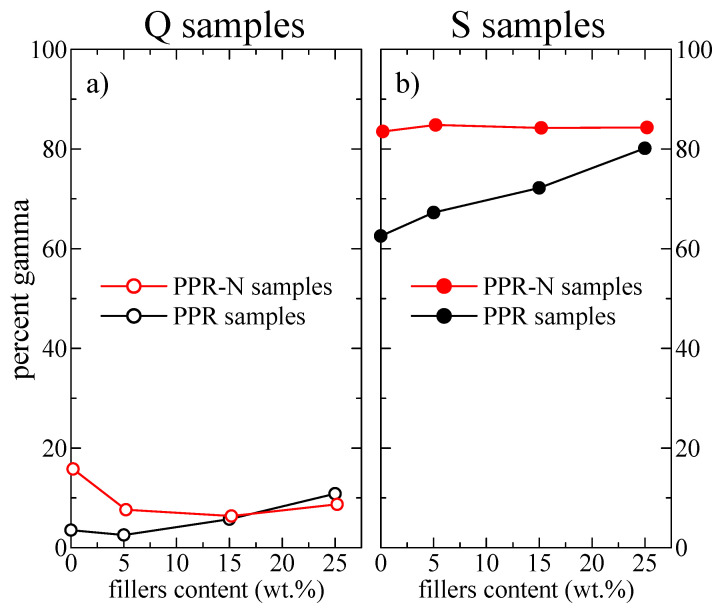
Variations in the percentages of γ modifications as functions of the filler content (DSF and Millad nucleant): (**a**) Q samples; (**b**) S samples.

Figure 3a,b show the variations in the percentages of γ modifications, which were obtained using Equation (2), as functions of the filler content (DSF and Millad nucleant) for the Q and S thermal treatments, respectively. Focusing attention on the PPR Q specimens, the γ percentage is rather small in virgin PPR (around 4%), although it increases with the DSF content in the sample to around 11% for specimen PPR-D25. The initial γ content for the Q specimen of sample PPRN, which was nucleated with Millad, can be seen to be significantly higher at around 16%, although the percentage clearly decreases with increasing DSF content, in such a way that similar values can be seen for samples PPR-D25 and PPRN-D25.

The fact that the γ proportion decreases with increasing DSF content in the PPRN-Q samples is attributed to the reduction of the nucleation ability caused by the presence of DSF (see below), since the general trend is that the γ percentage clearly increases with the nucleation ability.

The γ content was considerably higher for the slowly cooled S samples, as observed in Figure 3b. Again, the γ percentage was higher for sample PPRN (around 84%) compared with the 62% for virgin PPR; however, the γ fraction remained approximately constant with increasing DSF content in the nucleated samples, while it clearly increased in the non-nucleated samples, so that again samples PPRN-D25 and PPR-D25 displayed rather similar γ contents.

It is well known that sorbitol derivatives, such as Millad, exhibit considerable potential as nucleating agents and additionally act as excellent clarifiers when incorporated (usually in small amounts) into a polyolefin matrix; thus, they are known to increase the nuclei density, leading to very small spherulites, so that the transparency is considerably improved [50]. Moreover, they enhance the γ modification in PPR copolymers [51].

The results in Figure 3 also indicate that the DSF cellulose fiber considerably increases the γ content. Moreover, one reason for the constancy of the γ fraction in the S specimens of PPR-N may be related to the fact that the maximum value of the γ content [52] able to be developed in this specific matrix under these crystallization conditions could have been reached.

**Figure 4:**
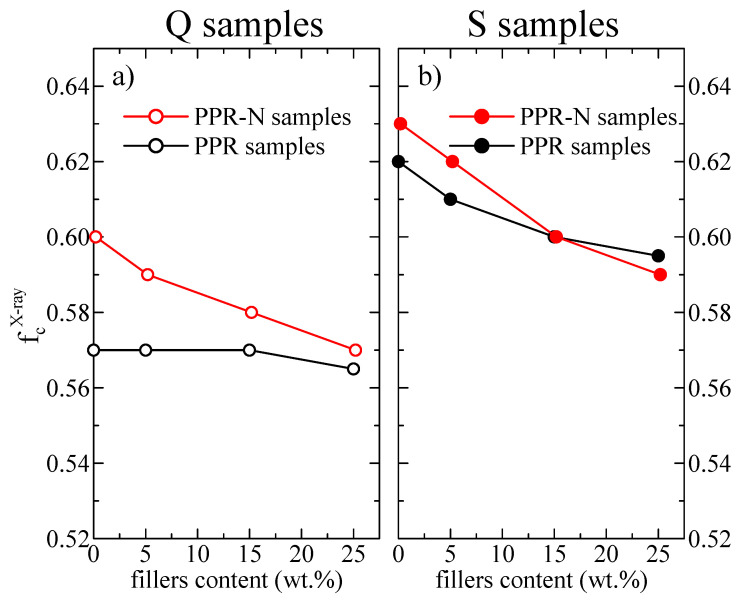
Results of the X-ray crystallinity as a function of the filler content (DSF and Millad nucleant): (**a**) Q samples; (**b**) S samples.

Another feature that can be derived from the diffractograms in Figure 2 is the determination of the X-ray crystallinity. As mentioned in the Materials and Methods, this was performed by subtracting the corresponding amorphous component taken from the profile of a totally amorphous elastomeric PP sample. The X-ray crystallinity results as a function of the fillers content (DSF and nucleated with Millad additive) are shown in Figure 4a,b for the Q and S thermal treatments, respectively. For the non-nucleated Q samples, the crystallinity is approximately constant at around 0.57. In contrast, sample PPRN-Q exhibits higher crystallinity (0.60), although this parameter decreases with the DSF content, with the values approaching those of the non-nucleated samples at high DSF compositions.

Again, these distinct behaviors are attributed to changes in the nucleation ability caused by the presence of DSF.

The behavior is different for the S samples: the initial crystallinity for the specimens without DSF is significantly higher than the one for the Q counterparts, although it decreases in both series of samples with increasing DSF content (from 0.62–0.63 to around 0.59). The estimated error in these results is in the order of 0.02.

### 3.3. DSC Results

Considering the previous X-ray outcomes, DSC experiments were performed with several objectives—as attempts to simulate the Q and S thermal treatments and to study the influence of DSF and Millad nucleant on the thermal transitions of PPR and on its nucleation ability.

**Figure 5:**
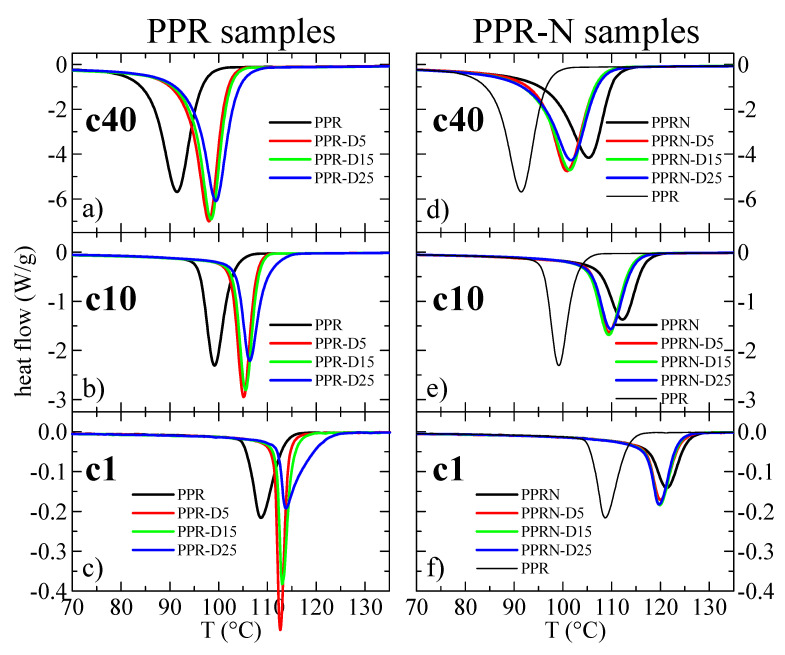
DSC cooling curves for the different samples: (**a**–**c**) curves from cooling of the melt at 40, 10, and 1 °C/min, respectively, for the PPR samples; (**d**–**f**) curves from cooling of the melt at 40, 10, and 1 °C/min, respectively, for the PPR-N samples. The curves for sample PPR are also included in the diagrams for the nucleated samples as references in order to better quantify the nucleation ability. All curves were normalized to the actual polymer content in the sample.

**Figure 6:**
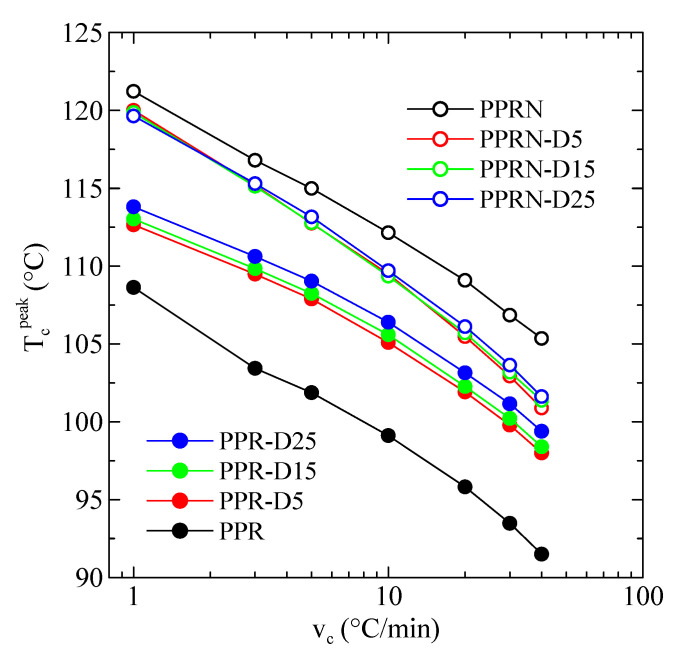
Variations in the peak crystallization temperature with the cooling rate for the different samples.

As mentioned in the Materials and Methods, it is not possible to use cooling rates higher than 40 °C/min, since the calorimeter loses temperature control before the crystallization exotherm occurs; therefore, different cooling rates from the melt were tested, ranging from 40 to 1 °C/min. Figure 5 show the curves from cooling from the melt at 40, 10, and 1 °C/min for the PPR and PPR-N samples. The outstanding nucleation ability of both the Millad nucleant and the DSF can be readily observed. In fact, Figure 6 represents the variations in the peak crystallization temperature with the cooling rate for the different samples.

An almost logarithmic variation was obtained in all of cases and a decrease in the peak crystallization temperature of about 16–17 °C can be observed, passing from 1 to 40°/min. Evidently, this is an obvious consequence of the differences in cooling rate.

**Figure 7:**
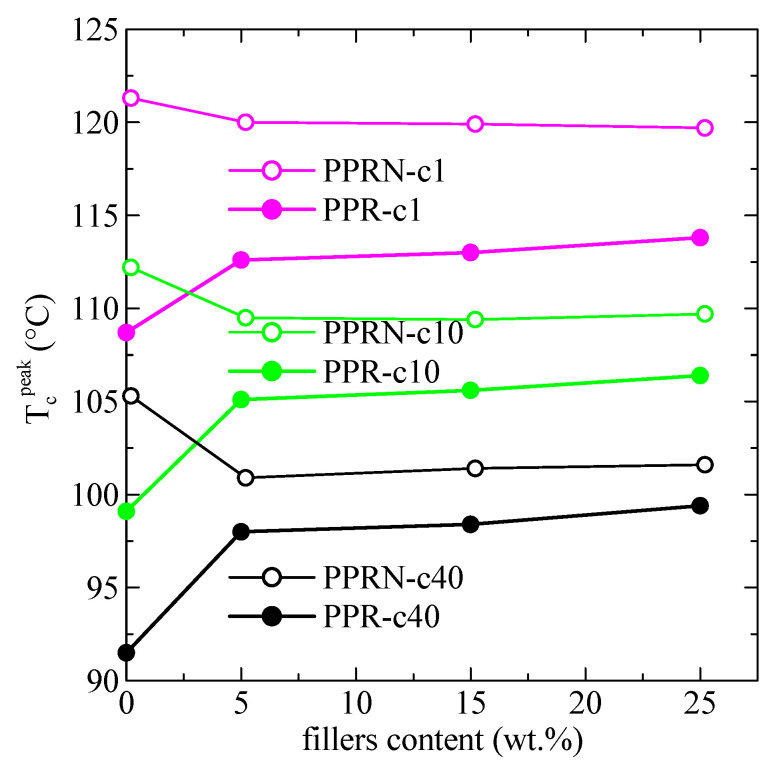
Variations in the peak crystallization temperature with the filler content (DSF and Millad) for the three indicated cooling rates, cx, with *x* in °C/min.

More relevant information can be deduced from Figure 7 when considering variations in the peak crystallization temperature with the filler content. Regarding the non-nucleated PPR samples, the observed behavior for all cooling rates is a remarkable increase in temperature when passing from PPR to PPR-D5, followed by a small but noticeable additional increase with increasing DSF content. For the nucleated PPRN samples, a well different behavior can be observed. A maximum value of T_c_^peak^ was obtained for the sample without DSF, which decreases initially with the DSF content, although small increases can again be noticed at higher DSF contents. The conclusion from these results is that an important nucleation ability is achieved from the addition of DSF.

**Figure 8:**
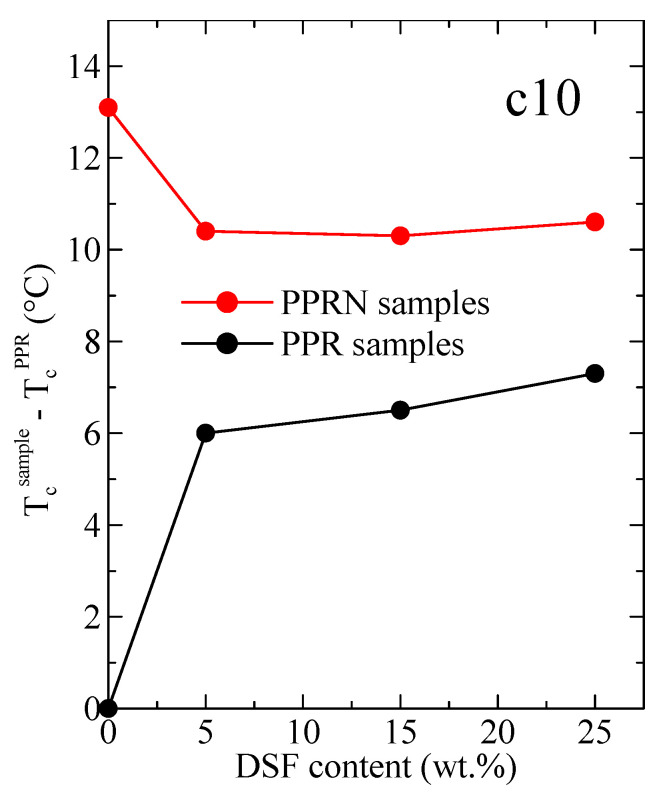
Variations with the DSF content in the differences between the peak crystallization temperature of a certain sample and that of the PPR specimen for the cooling rate of 10 °C/min.

This nucleation capability can be more clearly observed in Figure 8, representing the variation with the DSF content of the difference between the peak crystallization temperature of a certain sample and that of the neat PPR specimen at the cooling rate of 10 °C/min. An increase as large as 6 °C can be observed when passing from virgin PPR to PPR-D5, with further, much smaller increases for the higher DSF contents. These increases are considerably greater than the ones reported for composites of PP and DSF [9].

Comparison with the nucleated sample leads to the conclusion that the nucleation ability for a 25 wt.% DSF is approximately 55% of that for the Millad nucleant. This is quite a remarkable finding, as the extraordinary nucleation ability of Millad is well known (obviously, it has to be also considered that the Millad amount was only 0.2 wt.%). Another aspect shown in Figure 8 is that the addition of DSF to PPRN leads to an initial decrease in T_c_^peak^ of around 2.7 °C. It follows that the presence of DSF reduces the nucleation ability in relation to Millad alone, most probably arising from the contacts with the polymer matrix between Millad and DSF. Additionally, the adsorption of nucleation agents on the filler particles is another reason for the reduction in T_c_^peak^. On the other hand, the nucleation ability of these PPRN samples seems to increase slightly at higher DSF contents, as mentioned above.

**Figure 9:**
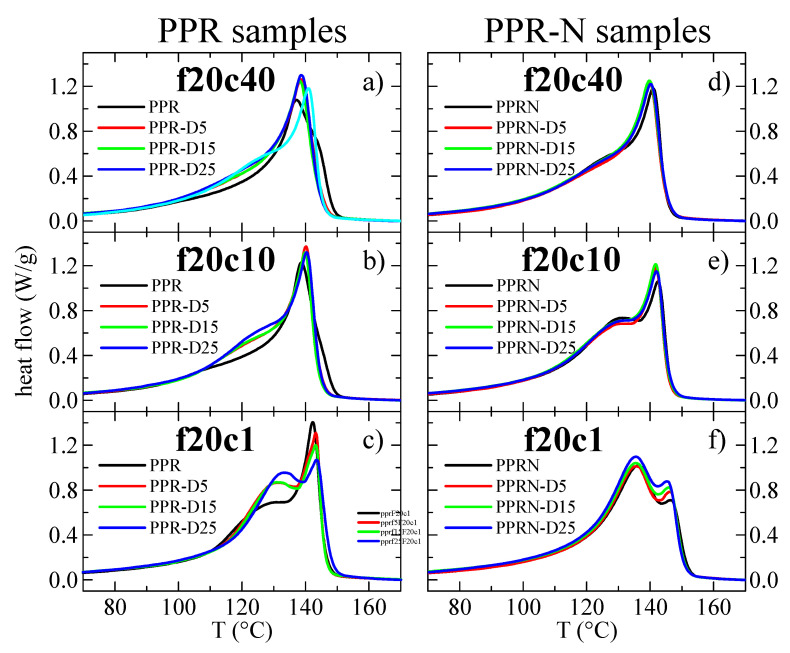
DSC melting curves for the different samples: (**a**–**c**) melting curves at 20 °C/min after cooling from the melt at 40, 10, and 1 °C/min, respectively, for the PPR samples; (**d**–**f**) melting curves at 20 °C/min after cooling from the melt at 40, 10, and 1 °C/min, respectively, for the PPR-N samples. All curves are normalized to the actual polymer content in the sample.

After the aforementioned cooling experiments, the subsequent melting curves were registered at a scanning rate of 20 °C/min. Figure 9 shows the melting curves after cooling from the melt at 40, 10, and 1 °C/min, respectively. After cooling at 40 °C/min, a small shoulder can be observed in the low-temperature region of the endotherms, which is more noticeable in the specimens with filler (either Millad or DSF). The shoulder increases greatly in intensity as the cooling rate decreases, and also with increasing DSF contents in the non-nucleated samples. In fact, it becomes predominant in all samples with filler, i.e., in the composites. This shoulder is known to arise from the melting of the orthorhombic γ modification [52,53,54], which melts at lower temperatures than the α form since the γ crystals are smaller in size, displaying less stability than the monoclinic ones, so that their melting temperatures are lower [52], even though they develop at higher temperatures.

Although it is difficult to deconvolute the melting curves in order to estimate the relative contents of the two polymorphs (especially because the profiles are non-symmetric), a rough determination of those contents can be made, which is easier when high proportions of γ crystals are present. For instance, the DSC results for samples PPR-D25 and PPRN-D25 after cooling at 1 °C/min were 78 and 82% of γ crystals, respectively. These values are in very good agreement with those derived from the X-ray results (see Figure 3b) for the S samples, where the cooling rate was also around 1 °C/min.

Another aspect deduced from the DSC curves in Figure 9 is that the melting temperatures of the composites are slightly higher than those of pristine PPR, particularly for the Millad nucleated samples. This is especially evident for the melting after cooling at 1 °C/min (Figure 9c,f), where differences as high as around 10 °C can be seen for the melting of the orthorhombic phase and around 4 °C in the melting for the monoclinic crystals. Obviously, the main reason for this is the previous crystallization at higher temperatures, owing to the very important nucleation ability of both the Millad nucleant and DSF.

### 3.4. Microhardness Results

Microhardness, MH, is a rather valuable mechanical parameter in polymer science. It is based on determining the ability of a certain material to plastic deformation, meaning that it provides a measure of strain on a local scale. Moreover, MH comprises a complex combination of different properties in relation to the viscoelastic behavior of the sample, including the modulus of elasticity, toughness, yield strength and strain hardening. There is a direct relation between the MH and elastic modulus [55,56]. In addition, MH experiments can be used as an easy and fast method for the determination of possible inhomogeneities derived in the processing of composite polymers and blends [36,57,58].

**Figure 10:**
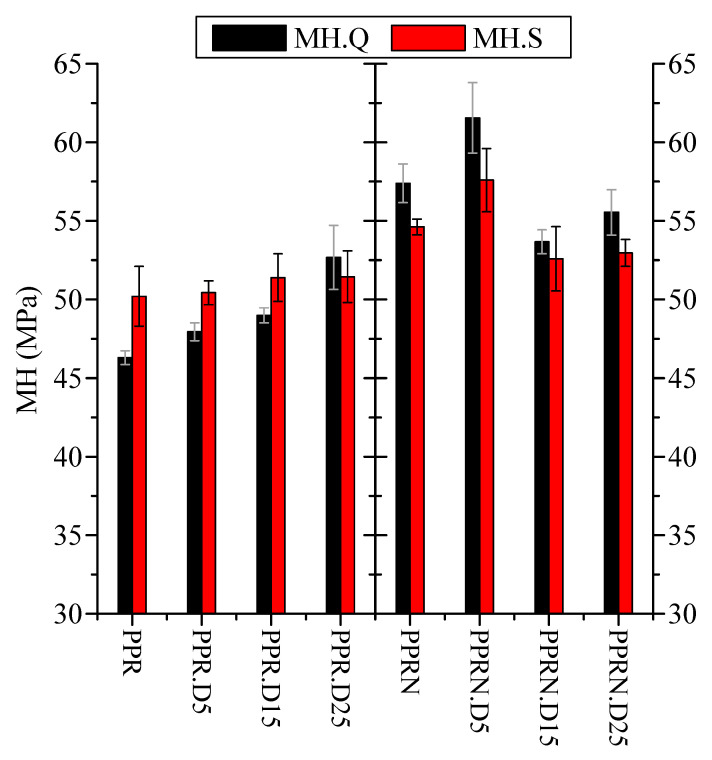
MH values for the different samples under the Q and S processing conditions.

The MH values for the different Q and S samples are shown in Figure 10. Focusing firstly attention on the non-nucleated samples, a clear increase in MH can be observed with the incorporation of DSF for the Q specimens, which is noticeably dependent on the content. Consequently, DSF particles play a reinforcement role in the PPR matrix. The increase is by about 15% when passing from PPR to PPR-D25. Similar improvements in the elastic modulus have been reported before for composites of PP and DSF [9].

The S specimens of the non-nucleated composites show a somewhat more complicated behavior, since the presence of DSF considerably modifies the PPR crystalline features in these samples. Details such as the degree of crystallinity, type and proportion of the polymorphs, as well as the lamellar thickness in the crystallites are also very important in the final rigidity exhibited by a polymeric material [39,57,58,59]. It should be mentioned that although crystallinity values for the S samples are higher than for the Q ones, their dependence upon DSF content is completely different (as seen in Figure 4). In the former a decrease is observed, while in the latter the crystallinity remains almost constant across the entire DSF interval. At the end, the combined effect of those two variables (DSF content and crystallinity) leads to rather constant MH values for these non-nucleated S samples.

Another difference is related to the contents in the polymorphs; most of the crystallites are monoclinic in the Q samples and orthorhombic in the S ones, as can be deduced from Figure 3. It has been described that the major presence of γ crystallites enhances the mobility in the amorphous regions compared with that promoted by the monoclinic crystals [59]. Accordingly, the γ crystals make a smaller contribution to the stiffness of the sample and then to microhardness than the monoclinic α crystals. All of these characteristics are responsible for the fact that the intrinsic reinforcement effect of DSF incorporation does not compensate for the reduction in the composites arising from the increase in γ content, furthermore showing a decrease in crystallinity.

In the nucleated composites, an initial increase in MH is observed at the lowest DSF load, independently of the thermal treatment applied. Nevertheless, the MH decreases at the higher contents. For a given processing condition (Q or S), the reduction in crystallinity, which is more important as DSF content is raised, again provokes this MH loss. Higher proportions of orthorhombic crystals in the S-nucleated composites lead to smaller MH values than those exhibited by the Q-nucleated specimens, in spite of the crystallinity being higher in the former than in the latter. Again, the presence of DSF is not able to balance the damage to the stiffness caused by the loss in PPR crystalline characteristics.

On the other hand, the homogeneity in the samples is found to be rather good, judging from the values of the standard deviation of the MH tests (see Materials and Methods); thus, the standard deviation, represented by the error bars in Figure 10, is always below 4%.

## 4. Conclusions

Several eco-composites were prepared from a polypropylene random copolymer and different amounts of date stone flour. The cellulosic fiber was silanized beforehand in order to reduce its hydrophilicity and to enhance the interfacial adhesion with the polymer. Additionally, similar composites were also obtained, including a sorbitol derivative (Millad 3988) as an effective nucleant. Films produced using two different thermal treatments (a slow cooling, S, and a rapid cooling, Q, from the melt) were analyzed.

The proper dispersion of the particles within the PPR composites, even at high loads of DSF, was revealed in the SEM images. The spherical shape of the particles was also identified, with an average size of around 10 microns.

Rapidly cooled specimens of the non-nucleated samples crystallized mainly under the monoclinic α form, while the orthorhombic γ polymorph was predominantly obtained in the slowly cooled samples, with the amount increasing with the DSF content. The γ crystals were also clearly favored by the presence of the sorbitol derivative nucleant. Furthermore, the degree of crystallinity in these non-nucleated Q composites remained rather constant with the DSF content. These crystallinity values were lower than those found in the non-nucleated S composites, for which a significant reduction in the crystallinity degree was noticed with increasing DSF composition.

DSC experiments at different cooling rates from the melt proved the outstanding nucleation ability of both the Millad nucleant and DSF; thus, increases in the peak crystallization temperature by as much as 6 °C were observed when passing from pristine PPR to PPR-D5 at a cooling rate of 10 °C/min, with smaller increases for higher DSF contents.

Comparison with the nucleated samples led to the conclusion that the nucleation ability for 25 wt.% DSF was approximately 55% of that for the Millad nucleant. It has to be considered, however, that the Millad content was only 0.2 wt.%. This is a rather remarkable finding, since the extraordinary nucleation ability of Millad is well known.

The subsequent melting experiments revealed that a shoulder occurred in the low-temperature region of the endotherms, which was more appreciable in the specimens containing filler (either Millad or DSF). The shoulder increased greatly in intensity as the cooling rate decreased and as the DSF contents in the non-nucleated samples increased, becoming predominant in all samples containing filler. This shoulder arises from the melting of the orthorhombic γ modification, which melts at lower temperatures than the α crystallites. Moreover, melting temperatures in the composites are slightly higher than those for neat PPR and particularly in those incorporating the Millad nucleant.

Increases of about 15% were found in the microhardness values of the rapidly cooled Q specimens when passing from PPR to PPR-D25. The behaviors for the slowly cooled specimens and all nucleated samples were somewhat more complicated, since there was an additional important factor to consider—changes in the PPR crystalline characteristics. The presence of DSF was not able to balance the damage caused in stiffness by the loss in PPR crystalline features. The MH measurements allow for the homogeneity of the DSF dispersion in the composites to be assessed through their low standard deviations, which are always kept below 4%.

All of these findings point out that silanized DSF can turn out to be a fairly good approach for use in polymeric eco-composites, taking advantage of the availability of lignocellulosic materials, which are otherwise wasted.

## Figures and Tables

**Table 1:** Chemical composition of date stone flour (wt.%).

Cellulose	Lignin	Hemicellulose	Ash	Extractable	Water
42.6	16.3	19.2	2.2	14.8	4.9

**Table 2:** Sample code nomenclature and formulations of the different composites.

Sample Code	Composition (wt.%)		
	PPR	DSF	Millad
PPR	100	0	0
PPR-D5	95	5	0
PPR-D15	85	15	0
PPR-D25	75	25	0
PPRN	99.8	0	0.2
PPRN-D5	94.8	5	0.2
PPRN-D15	84.8	15	0.2
PPRN-D25	74.8	25	0.2

## Data Availability

The data presented in this study are available on request from the corresponding author.
